# Effects of Phytase Transgenic Maize on the Physiological and Biochemical Responses and the Gut Microflora Functional Diversity of *Ostrinia furnacalis*

**DOI:** 10.1038/s41598-018-22223-x

**Published:** 2018-03-13

**Authors:** Xiao Hui Xu, Yinghui Guo, Hongwei Sun, Fan Li, Shuke Yang, Rui Gao, Xingbo Lu

**Affiliations:** Institute of Plant Protection, Shandong Academy of Agricultural Sciences, Shandong Key Laboratory of Plant Virology, Ji’nan, 250100 China

## Abstract

Transgenic maize hybrids that express the *Aspergillus niger phyA2* gene could significantly improve phosphorus bioavailability to poultry and livestock. However, little information has been reported about the effects of phytase transgenic maize on the Asian corn borer (ACB), *Ostrinia furnacalis* (Guenée). This study provides valuable information about the physiological, biochemical and gut microflora functional diversity changes of ACBs fed phytase transgenic maize. The weights, survival rates, *in vivo* protein contents, activities of two detoxification enzymes and three antioxidant enzymes of ACBs fed phytase transgenic maize exhibited no significant differences to those fed non-transgenic maize. Functional diversities of the gut microflora communities of ACBs were not affected by different fodder treatments, but significant differences were observed between different generations of ACBs. Our study provides useful information about the biochemical responses and gut microflora community functional diversities of ACBs fed phytase transgenic maize firstly and the results will help to assess the potential effects of phytase transgenic maize on other target and non-target arthropods in the future.

## Introduction

Phosphorus is an essential nutrient for most living organisms; including mammals, terrestrial insect herbivores, bacteria and algae^1–5^. In plants, phosphorus is mainly stored in oilseeds and cereal grains in the form of phytate^6^. However, phytate-P is absorbed at low levels in monogastric animals due to the absence of the phosphatase enzyme, phytase, in their digestive tract. To avoid the arrested growth and development of animals that is caused by phosphorus deficiency, two approaches have been developed. One approach is to add inorganic phosphate into animal feeds; however, this leads to severe phosphorus pollution of surface water and groundwater^7^. The other approach is to add phytase to the feed; however, this is prohibitively expensive. To reduce the cost, phytase transgenic maize was previously developed by Chen *et al*.^8^. Phytase activity in the kernels of this transgenic maize is approximately 50-fold higher than that of non-transgenic maize^8^. Therefore, using phytase transgenic maize as the major ingredient in animal feed could not only decrease feed costs but also be beneficial to the environment.

According to the ISAAA report in 2016, genetically modified (GM) maize was the second most popular GM crop in the world, with over 60.6 million hectares grown globally^9^. However, the debate about the safety of GM crops has never stopped. Before entering the market, a whole series of animal and environmental experiments must be carried out^10^. The potential effects on non-target organisms are considered to be one of the most important aspects of this assessment. The Asian corn borer (ACB), *Ostrinia furnacalis* (Guenée), is the main maize crop pest in Asia. The larvae of ACBs take the maize kernels as one of the major food. As the phytase was specifically expressed in the kernels of phytase transgenic maize^8^, ACBs are directly exposed to high concentrations of phytase proteins when they take kernels of phytase transgenic maize as the food source. Besides, ACBs could serve as prey that transfer potential effects to other organisms at higher trophic levels. Thus, we use ACB as an indicator for the potential ecological effects assessment of phytase transgenic maize on the non-target organisms.

Several aspects of the potential ecological effects of phytase transgenic maize on non-target organisms have been investigated. Using the pitfall trap method, the effects of phytase transgenic maize on the seasonal changes of carabid beetles were analysed^11^. In addition, the development and nutritional utilization of the ACB and *Helicoverpa armigera* were also tested by feeding the insects on phytase transgenic and non-transgenic maize^12^. The combined results from these studies have shown that phytase transgenic maize has no obvious impact on the seasonal changes of carabid beetles, or on the development and nutritional utilization of the ACB and *H. armigera* species^11,12^.

The functional diversity of a microbial community is its potential activities, including numbers, types and rates, in utilizing a suite of substrates. Understanding the functional diversity of a microbial community is an essential prerequisite to uncover the roles of microbial communities in different environments. Community-level physiological profiles (CLPPs) can be useful for evaluating the functional diversities of microbial communities and they have been widely used to differentiate microbial communities^13^. Biolog EcoPlates, which contain a large number of ecologically relevant structurally diverse carbon substrates, are commonly used to determine the CLPPs of culturable bacteria^14^. In comparison with alternative approaches, Biolog EcoPlates method has many advantages, such as simplicity, convenience and one experiment could produce adequate information about functional diversity of bacteria. Many published studies have used Biolog EcoPlates to determine the functional diversities of microbial communities^13,15,16^.

Up to date, only a few parameters have been compared at or before the early 3^rd^ instar between ACB larvae fed phytase transgenic maize and control maize^12^. These include larval survival rate, duration of the 1^st^ and the 2^nd^ instar larvae, fresh weight and nutritional utilization. These indices at the early stage of ACB development could not reflect the full impacts of phytase transgenic maize on ACBs. In this study, the effects of phytase transgenic maize on ACB were investigated more comprehensively. The survival rate and fresh weight of ACBs at the beginning of the 5^th^ instar were analysed. Importantly, the biochemical effects and the functional diversities of gut microbial communities of ACBs fed phytase transgenic maize were investigated firstly in this study. The results contribute significantly to elucidate the biological effects of phytase transgenic maize on ACBs and provide new clues to evaluate the effects of GM crops on other arthropods.

## Results

### Effects of different maize lines on the growth and development of ACB larvae

At the beginning of the 5^th^ instar, ACB larvae fed phytase transgenic maize and control lines (Liyu 35 and Zhengdan 958) were collected to calculate survival rate and fresh weight. As shown in Table 1, both the weights and survival rates of larvae fed phytase transgenic maize kernels and non-transgenic maize kernels showed no significant differences in each generation (*P* value > 0.05). Moreover, the two indices were similar in three generations under all fodder treatments (Supplementary Table 1a and b). The results suggest that phytase transgenic maize does not negatively affect either the survival rate or weight of the ACB.

**Table 1:** Comparison of survival rates and weights for three generations of Asian corn borer larvae.

Gen	Measurement	phytase transgenic maize	Liyu 35	Zhengdan 958
The 1^st^ Gen	Survival Rate (%)	86.00 ± 1.73a	85.00 ± 2.65a	87.00 ± 2.65a
	Weight (mg)	81.57 ± 15.79a	85.23 ± 19.67a	78.20 ± 16.64a
The 2^nd^ Gen	Survival Rate (%)	88.00 ± 2.00a	86.33 ± 1.53a	88.33 ± 1.15a
	Weight (mg)	78.77 ± 6.31a	77.70 ± 6.62a	81.2 ± 4.21a
The 3^rd^ Gen	Survival Rate (%)	85.00 ± 2.00a	83.33 ± 1.53a	84.67 ± 2.52a
	Weight (mg)	84.77 ± 16.09a	80.47 ± 13.70a	83.17 ± 13.10a

Gen, generation.

### ACB biochemical responses with different fodder treatments

The total protein content and enzyme activities of glutathione S-transferase (GST), acetylcholinesterase (AChE), catalase (CAT), peroxidase (POD) and superoxide dismutase (SOD) were tested to determine the biochemical responses of the ACBs after feeding with either phytase transgenic or non-transgenic maize.

As shown in Table 2, the ACB larvae in each generation fed with each of the three kinds of fodder showed similar total protein contents, ranging from 97.24 mg/mL to 106.49 mg/mL. The total protein content of the larvae in three generations with each fodder treatment showed no significant difference (*P* value > 0.05) (Table 2 and Supplementary Table 1c).

**Table 2:** Comparison of biochemical responses in three generations of the Asian corn borer.

Gen	Fodder	Total protein content (mg/ml)	CAT activity (U/g pro)	POD activity (U/g pro)	SOD activity (U/mg pro)	GST activity (U/mg pro)	AChE activity (U/g pro)
The 1^st^ Gen	phytase transgenic maize	102.79 ± 4.76a	81.79 ± 8.85a	73.46 ± 4.12a	403.81 ± 24.44a	3.24 ± 0.13a	17.15 ± 1.18a
	Liyu 35	105.48 ± 10.91a	88.81 ± 7.80a	68.19 ± 7.17a	388.65 ± 25.56a	3.23 ± 0.08a	15.28 ± 1.72a
	Zhengdan 958	97.24 ± 13.72a	77.19 ± 5.99a	75.45 ± 8.94a	428.03 ± 66.17a	3.20 ± 0.07a	16.54 ± 1.24a
The 2^nd^ Gen	phytase transgenic maize	103.75 ± 6.17a	77.52 ± 2.09a	64.63 ± 4.77a	453.47 ± 31.93a	3.32 ± 0.30a	17.03 ± 1.28a
	Liyu 35	105.31 ± 5.26a	81.61 ± 11.40a	69.43 ± 1.38a	450.13 ± 32.57a	3.22 ± 0.33a	18.60 ± 0.11a
	Zhengdan 958	102.03 ± 8.02a	83.31 ± 9.39a	71.07 ± 6.98a	457.72 ± 46.43a	3.33 ± 0.39a	17.29 ± 1.34a
The 3^rd^ Gen	phytase transgenic maize	103.50 ± 11.22a	82.26 ± 13.81a	63.61 ± 5.56a	437.04 ± 44.55a	3.26 ± 0.44a	20.47 ± 0.91a
	Liyu 35	106.49 ± 10.54a	81.48 ± 13.03a	67.95 ± 4.62a	444.28 ± 55.01a	3.12 ± 0.29a	19.90 ± 0.85a
	Zhengdan 958	102.15 ± 8.54a	89.23 ± 1.21a	68.70 ± 3.22a	435.21 ± 55.75a	3.26 ± 0.23a	19.79 ± 0.45a

Gen, generation.

Larvae from different generations showed similar CAT and POD activities, with 77.19–89.23 U/g protein (pro) and 63.61–75.45 U/g pro, respectively (Table 2). The SOD activities in three generations of larvae were much higher than the other two protective enzymes, CAT and POD, which reached more than 380 U/mg pro (Table 2). The activities of the two detoxification enzymes (GST and AChE) fell into 3.12–3.33 U/mg pro and 15.28–20.47 U/g pro, respectively (Table 2). Overall, the activities of both the detoxification and protective enzymes in the three larval generations and with the three different fodder treatments did not produce any significant differences (*P* value > 0.05) (Table 2, Supplementary Table 1d–h). These results show that the phytase transgenic maize does not significantly affect the physiological properties of ACB larvae.

### Functional diversity of gut microflora of the ACB fed different maize lines

#### Dynamic changes in the gut microbial community in three generations of ACB

**Figure 1:**
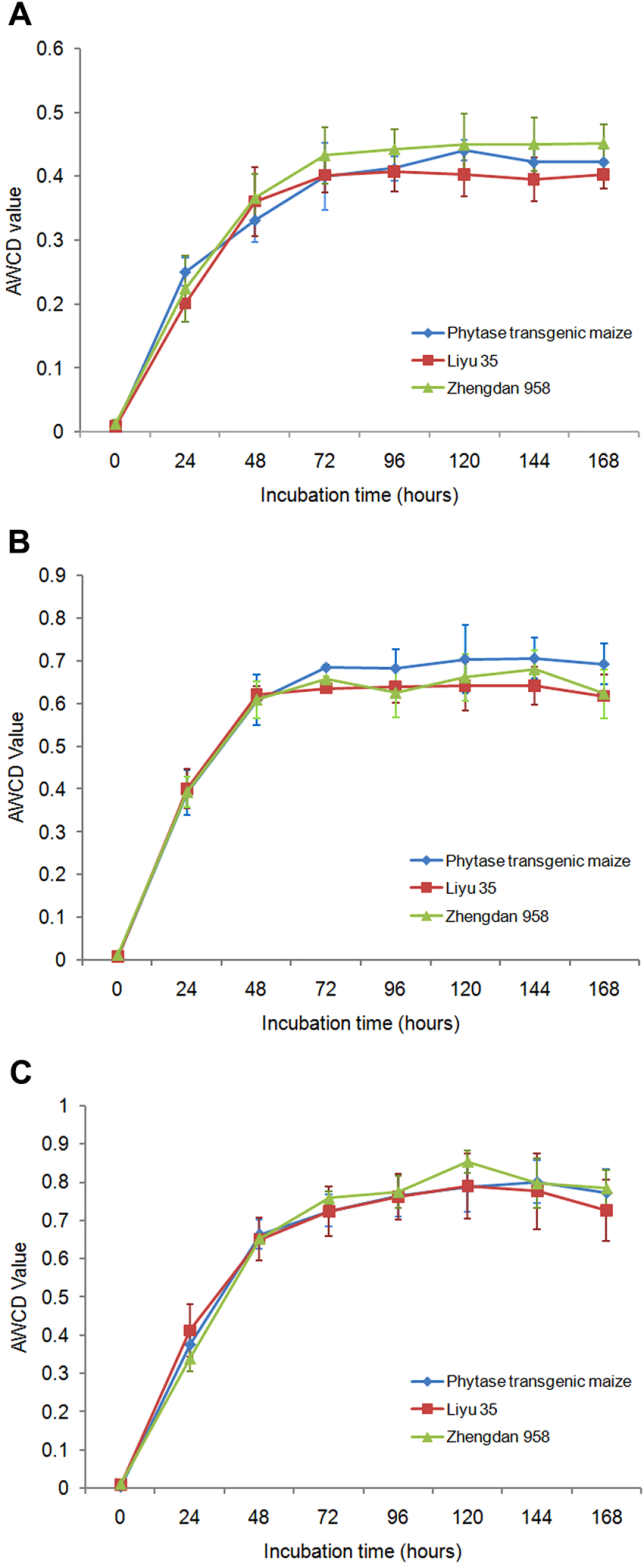
Change in the average well color development (AWCD) of gut microflora in three generations of Asian corn borers (ACBs) with different fodder treatments. (**A**) The first generation. (**B**) The second generation. (**C**) The third generation.

The average well color development (AWCD) value is an important index of overall metabolic activity for a microbial community^17,18^. With more species and greater abundances of gut microflora, more types and greater amounts of carbon sources will be utilized in the plate cells. In general, AWCD of a certain microbial community correlates with the growth of the cultured microbes. Two phases of AWCD changes were observed in this study, a linear increasing stage and a stationary phase (Fig. 1). During the first 72 h, microbes grew rapidly and correspondingly, the carbon sources were also rapidly utilized. Later, the abundances of microbes stopped increasing and the carbon sources were thereafter utilized at a steady state. As shown in Fig. 1, the changes of AWCDs of the ACBs in the three generations were similar. ACBs fed phytase transgenic maize in each generation did not exhibit significantly different AWCDs between the three different fodder treatments, at the linearly increasing stage or at later time points. However, different generations of ACBs fed the same fodder showed significant differences in the AWCD values at each time point except 0 h (Supplementary Table 2). Two way ANOVA analyses showed the interaction of the two variances (fodder and generation) had no significant impact on the changes of AWCD values at each time point (Supplementary Table 2). These results show that phytase transgenic maize has no obvious effects on the quantity and structure of the ACB gut microbial community.

#### Carbon substrates utilized by the gut microbial communities in three generations of ACBs fed with three fodders

**Figure 2:**
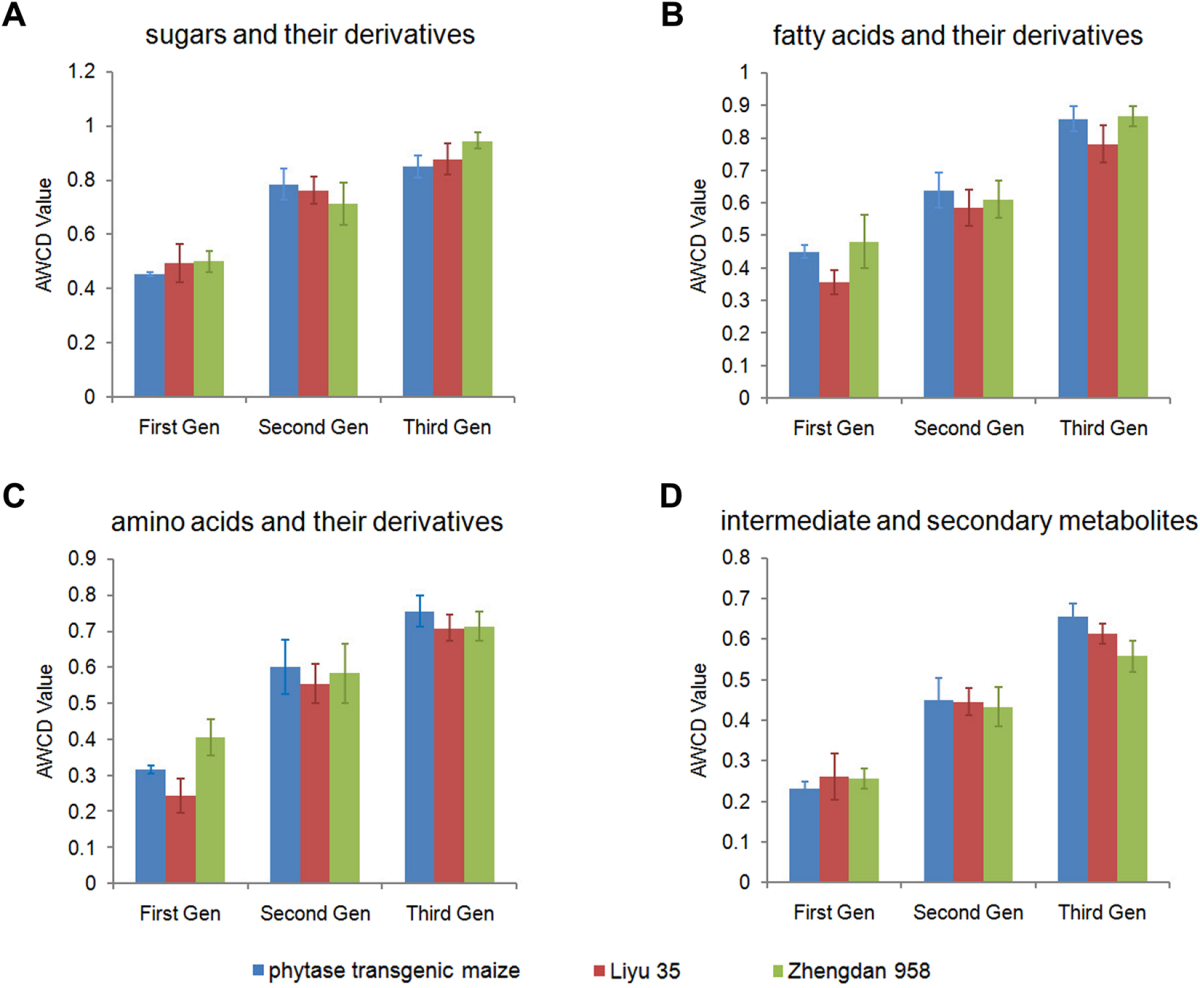
The utilization of four types of carbon sources by gut microflora from three generations of Asian corn borers. (**A**) Sugars and their derivatives. (**B**) Fatty acids and their derivatives. (**C**) Amino acids and their derivatives. (**D**) Intermediate and secondary metabolites. Gen, generation.

As shown in Fig. 1, the gut microflora used the carbon sources at a steady state after 96 h incubation; therefore, utilization of carbon sources at this time could reflect the difference of individual carbon source utilization by ACBs under different fodder treatments. The 2^nd^ and the 3^rd^ ACB generations utilized more carbon substrates than the 1^st^ generation (Fig. 2 and Supplementary Table 3). For each generation, sugars and their derivatives seemed to be the favourite carbon source for the gut microbial community. Moreover, the utilization of fatty acids, amino acids and their derivatives are relatively lower and the utilization of intermediate and secondary metabolites was the lowest. For each generation of ACBs fed three fodders, the utilization of each kind of carbon substrate showed no significant difference (Supplementary Table 3).

#### Diversity index analysis of the gut microbial communities in three generations of ACBs

Three indices, including Shannon’s diversity index (H), Simpson index (D) and McIntosh index (U), were calculated to evaluate the species richness, dominance and homogeneity of the gut microbial communities of ACBs under different fodder treatments^19–21^, respectively.

Duncan’s new multiple range test of data collected in each generation revealed that the three indices H, D and U of ACB fed phytase transgenic maize were similar to those fed control maize (Table 3). However, all the three indices showed great significant differences between different generations (Supplementary Table 4). Taking into consideration of both fodder and generation as two variables, the significance of the interaction of the two factors were weaker, only U showed significant differences (Supplementary Table 4). Thus, it can be concluded that phytase transgenic maize does not significantly affect the gut microbial community species’ richness, dominance and homogeneity in the ACB in one generation, but generation seemed to make a great contribution to determine H, D and U.

**Table 3:** Comparison of the gut microbial community diversity index for three generations of Asian corn borer.

Gen	Fodder	H	D	U
The 1^st^ Gen	phytase transgenic maize	3.22 ± 0.05a	0.96 ± 0.00a	2.36 ± 0.02a
	Liyu 35	3.18 ± 0.05a	0.96 ± 0.00a	2.42 ± 0.31a
	Zhengdan 958	3.25 ± 0.07a	0.96 ± 0.00a	2.60 ± 0.16a
The 2^nd^ Gen	phytase transgenic maize	3.32 ± 0.00a	0.96 ± 0.00a	3.90 ± 0.28a
	Liyu 35	3.32 ± 0.00a	0.96 ± 0.00a	3.73 ± 0.27a
	Zhengdan 958	3.33 ± 0.01a	0.96 ± 0.00a	3.58 ± 0.37a
The 3^rd^ Gen	phytase transgenic maize	3.36 ± 0.00a	0.96 ± 0.00a	4.60 ± 0.12a
	Liyu 35	3.35 ± 0.00a	0.96 ± 0.00a	4.49 ± 0.22a
	Zhengdan 958	3.35 ± 0.02a	0.96 ± 0.00a	4.61 ± 0.15a

Gen, generation.

#### Principal Component Analysis (PCA) of gut microbial community metabolism in three generations of the ACB

**Figure 3:**
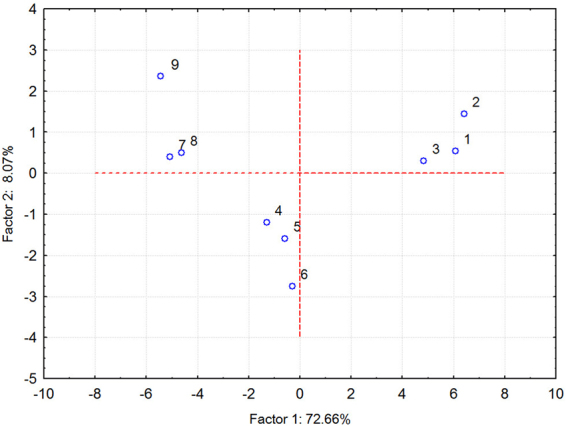
The principal component analysis of the carbon substrates used by the gut microflora of three generations of Asian corn borer (ACB). 1, 2 and 3 represent the 1^st^ generation of ACBs fed phytase transgenic maize, the parental strain Liyu 35 and the non-GM maize Zhendan 958, respectively; 4, 5, 6 represent the 2^nd^ generation of ACBs fed phytase transgenic maize, Liyu 35 and Zhendan 958, respectively; 7, 8, 9 represent the 3^rd^ generation of ACBs fed phytase transgenic maize, Liyu 35 and Zhengdan 958, respectively.

PCA analysis converts a set of observations of possibly correlated variables into a set of values of linearly uncorrelated (orthogonal) variables and the metabolic features of different microbial communities can be presented as relative positions in a 2-D figure^22^. Based on the AWCD values after 96 h incubation, the single carbon source utilization patterns of the ACB intestinal microbial communities after the three different fodder treatments were analysed by PCA. In total, eight principal components (PCs) were extracted. The proportion and cumulative proportion of the eight PCs were shown in Table 4. PC1 accounted for the largest proportion of total variation (72.66%), while PC2, PC3 and PC4 accounted for similar variances to one another (8.07%, 6.45% and 5.96%, respectively). Therefore, the metabolic features of the microbial community could be represented well by the first four principal components, PC1–PC4. As shown in Fig. 3, different generations of ACBs were distributed in different areas of the 2-D figure. The 1^st^ generation of ACBs that were fed phytase transgenic and non-transgenic maize kernels were distributed on the positive axis of PC1, while the 2^nd^ and the 3^rd^ generations of ACBs that were fed the three different fodders were distributed on the negative axis (Fig. 3). ACBs fed the same fodder in all three generations did not form obvious groups, but ACBs in three generations were clustered in three groups (Fig. 3). Twenty-eight carbon substrates were primarily concentrated on PC1, with the exception of L-arginine (mainly concentrated on PC4), 2-hydroxy benzoic acid (mainly concentrated on PC2) and phenylethylamine (mainly concentrated on PC1 and PC2) (Table 5). These results indicated that the gut microbial communities of ACBs fed phytase transgenic maize have similar carbon utilization patterns to communities from ACBs fed non-transgenic maize.

**Table 4:** The proportion and cumulative proportion of the eight principal components.

Principal Component	Total variance (%)	Cumulative %
PC1	72.66	72.66
PC2	8.07	80.73
PC3	6.45	87.18
PC4	5.96	93.14
PC5	3.30	96.44
PC6	1.68	98.12
PC7	1.36	99.48
PC8	0.52	100.00

**Table 5:** Four types of carbon substrates loaded on the former four principle components.

category	Carbon substrate	PC1	PC2	PC3	PC4
sugars and their derivatives	ß-Methyl-D-Glucoside	−0.95	0.26	−0.02	−0.01
	D-Galactonic Acid y-Lactone	−0.86	0.29	0.11	−0.09
	D-Xylose	−0.91	−0.25	0.10	0.24
	D-Galacturonic Acid	−0.98	−0.13	−0.02	−0.03
	i-Erythritol	−0.78	0.10	−0.55	0.07
	D-Mannitol	−0.76	0.22	0.34	0.38
	α-Cyclodextrin	−0.93	−0.17	0.14	0.10
	N-Acetyl-D-Glucosamine	−0.94	0.21	0.15	0.03
	Glycogen	−0.84	−0.51	−0.09	0.03
	D-Glucosaminic Acid	−0.94	−0.12	0.03	0.21
	D-Cellobiose	−0.91	0.12	0.31	0.19
	α-D-Lactose	−0.78	−0.18	0.52	0.10
fatty acids and their derivatives	Pyruvic Acid Methyl Ester	−0.96	0.04	0.04	0.14
	Tween 40	−0.84	0.38	−0.12	−0.22
	Tween 80	−0.96	0.14	−0.10	−0.07
	γ-Hydroxybutyric Acid	−0.65	−0.16	−0.71	−0.09
	Itaconic Acid	−0.97	0.08	−0.08	0.13
amino aicds and their derivatives	L-Arginine	−0.28	−0.30	0.20	−0.88
	L-Asparagine	−0.95	0.12	0.08	0.05
	L-Phenylalanine	−0.87	−0.25	−0.28	0.07
	L-Serine	−0.99	0.01	0.01	−0.02
	L-Threonine	−0.95	−0.18	−0.09	0.05
	Glycyl-L-Glutamic Acid	−0.93	0.01	−0.35	0.10
intermediate and secondary metabolites	2-Hydroxy Benzoic Acid	−0.02	−0.65	−0.28	0.22
	4-Hydroxy Benzoic Acid	−0.95	0.11	−0.07	−0.08
	Glucose-1-Phosphate	−0.94	−0.09	0.26	0.17
	α-Ketobutyric Acid	−0.69	−0.26	0.23	−0.58
	Phenylethylamine	−0.58	−0.74	0.06	−0.14
	D,L-α- Glycerol Phosphate	−0.92	−0.03	0.24	−0.15
	D-Malic Acid	−0.74	0.45	−0.13	−0.39
	Putrescine	−0.85	0.39	−0.22	−0.16

## Discussion

With the advent of plant biotechnology, increasing numbers of transgenic crops with various properties, such as insect resistance, herbicide tolerance and improved quality, have been developed and planted in several countries. The potential environmental impacts of these GM crops should be investigated before they enter the market. Previous studies have mainly focused on the environmental risks of insect-resistant transgenic crops expressing *Bacillus thuringiensis* (Bt) toxins, protease inhibitors and lectins^23–26^. Phytase transgenic maize is the first transgenic maize developed in China that has obtained a security certificate^8^. The use of phytase transgenic maize can not only improve the phytate-P utilization of livestock, but can also reduce the cost of fodder production. Although phytase is only over-expressed in the kernels of phytase transgenic maize and these plants do not carrying the insect-resistant trait, they may still affect the growth and development of any living organisms. These may include terrestrial insect herbivores, bacteria and algae; because phosphorus is necessary for the growth and development of all these organisms^2,3,5,27^. Moreover, the nutritional characteristics of crops may be altered after genetic transformation^28^ and this might directly or indirectly affect the fitness of organisms that use these plants as food sources. Consequently, evaluation of the potential effects of phytase transgenic maize on non-targeted organisms is important. The ACB is a major threat to maize production in Asia and mainly feeds on maize kernels at its larval stage. It could be used as an indicator for the assessment of the ecological effects of GM crops on non-target organisms. This study provides the first detailed information on the physiological and biochemical responses, as well as the changes in the functional diversity of gut microflora, in ACBs consuming phytase transgenic maize.

Several studies have been carried out to investigate the impacts of phytase transgenic maize on the target and non-target organisms. It was found that phytase transgenic maize has no adverse effects on terrestrial vertebrates; including pigs^29^, laying hens^30^ and roosters^31^. However, studies have shown that the growth and development rates of some insects, including *Choristoneura occidentalis*, *Acheta domesticul* and *Manduca sexta*, are accelerated when they are fed diets that contain high concentrations of phosphorus^3,32,33^. The published papers concentrated on the potential effects of phytase transgenic maize on herbivorous insects, including ACB, *H. armigera* and carabid beetles showed no significant differences between phytase transgenic maize and its isogenic maize^11,12^. Diets contain high concentrations of phytase transgenic maize did not affect the survival, fresh weight, nutrition utilization and the duration of the 1^st^ and the 2^nd^ instars of ACBs and *H. armigera*^12^. To fully appreciate the effects of phytase transgenic maize on ACBs, we examined multiple physiological, biochemical and gut microflora parameters in ACBs fed diets contain phytase transgenic maize at the 4^th^ or the 5^th^ instar. Three generations were used for each study to guarantee repeatability. In agreement with the previous study^12^, the survival rates and fresh weights of ACBs were unaffected with consumption of phytase transgenic maize (Table 1), which indicate phytase transgenic maize has no obvious direct toxicity to ACBs. In addition, we also found no significant differences in two detoxification enzymes (GST and AChE) and three antioxidant enzymes (CAT, POD and SOD) across three generations of ACBs fed phytase transgenic maize, in comparison with the controls (Table 2). Together, these data suggest that phytase transgenic maize has no measurable toxicity to ACBs.

The gut microbial communities of insects play important roles in the growth and development of their hosts. They aid in the digestion of heavy metals, protect hosts from parasites and pathogens, modulate immune responses and contribute to inter- and intraspecific communication. Changes in gut microbial communities could reflect the health status of the insect’s digestive system^34^. Biolog EcoPlate is a powerful tool in evaluating the functional diversity of a microbial community since functional changes of microbial communities could be reflected rapidly and sensitively. AWCD is an important indicator of microbial community activity and reflects the ability to utilize available carbon sources. The similar AWCD values obtained for ACB gut microbial communities under different fodder treatments in each generation indicate that phytase transgenic maize has no obvious effects on the total microbial activity. The three diversity indices, H, D and U, reflect different aspects of a microbial community. H represents the richness and evenness, D emphasizes the common species in a community and U measures microbial community species homogeneity. This study revealed that there were no significant differences in H, D or U between ACBs fed phytase transgenic maize and control lines in each generation (Table 3). However, H, D and U values changed great significantly between different generations (Supplementary Table 4). Consistent with this result, PCA analysis revealed that ACBs fed different fodders in one generation clustered into the same group, while different generations of ACBs clustered into different groups (Fig. 2). As the 2^nd^ and the 3^rd^ generations of ACBs were the offspring of the 1^st^ generation, we propose that consuming the same food over multiple generations may have a cumulative effect on the functional diversity of the ACB gut microbial community. Although some differences were found in the ACB gut microbial communities in different generations, we did not observe any differences between the gut microbial communities of ACBs fed phytase transgenic maize and control lines in a single generation (Table 3). The effects of phytase transgenic maize on the ecology of microorganisms in the rhizosphere have been tested. Consistent with our results, the microbial community structure in the phytase transgenic maize rhizosphere was similar to that of control maize lines^35^.

In summary, at least in the short term, phytase transgenic maize does not appear to disrupt the normal physiological activities, biochemical responses, or gut microbial community functional diversity in the ACB. This study serves as strong foundation for the evaluation of transgenic plant safety and supports the continued propagation of phytase transgenic maize in the environment.

## Methods

### Plant Materials

Phytase transgenic maize 10TPY005, Liyu 35 (the non-transgenic parent of 10 TPY005) and Zhengdan 958 (non-GM maize) were grown in the transgenic experimental station of the Shandong Academy of Agricultural Sciences in Jinan, China. Before tassels began shedding, the tassels and husks were bagged to prevent cross-pollination and pathogen invasion. Approximately 3 d after silks emerged from the husks, they were self-pollinated to produce pure seeds. The immature maize kernels were collected at 15 d after artificial pollination. Once the maize kernels were mature, they were collected and then ground into a fine powder to make maize meal. The fodders (maize power 100 g, water 350 mL, agar 10 g, sorbic acid 0.5 g and methyl p-hydroxybenzoate 0.5 g) used for the larvae culture were prepared the same as that described by Zhang *et al*.^12^ and were made into several thick hollow cylinders. After cooling and solidification, the fodders were vacuum freeze dried and sealed in plastic bags at −80 °C until use. To guarantee the activity of phytase, the fodders at room temperature were placed into petri dishes and saturated with maize juice that was prepared by homogenization of immature maize kernels. The saturated fodders were used as the food sources for the ACB larvae.

### The culture of ACB

ACB larvae were acquired from the Institute of Plant Protection, Shandong Academy of Agricultural Sciences. Fodders containing phytase transgenic maize, Liyu 35 or Zhengdan 958 were cut into small cubes (~1.0 g) and then cubes were individually placed into wells of a 24-well cell culture plate (Tianyu, Tianjin, China). Neonates were transferred individually to the surface of fodder cubes with a soft brush. Then the cell culture plates were sealed with lids.

After pupation, the ACB was transferred into a 200 mL beaker at 26 ± 1 °C, with a 70 ± 10% relative humidity and a 16 h light: 8 h dark photoperiod. The inner surface of the beaker was covered with A4 paper onto which the ACBs could lay their eggs and the rims of the beakers were sealed with pieces of cotton with a 0.25 mm sieve diameter. Adult female and male ACBs were paired for breeding and were fed with immature maize kernels and supplemented with a cotton ball that had been pre-soaked in a 3% sugar solution. The eggs produced were promptly removed until the adults died^12^.

### Growth and developmental measurements of ACB

Three generations of ACBs fed phytase transgenic maize and non-transgenic maize (Liyu 35 and Zhangdan 958) were used in this experiment, with the 2^nd^ and the 3^rd^ generations being offspring of the 1^st^ generation.

Both the survival rate and larval fresh weight were measured at the beginning of the 5^th^ instar stage. Each larva was gently touched with a writing brush tip and if there was no observed reaction, it was considered to be dead. Once a larva was died, it was removed immediately from the culture plate. The living ACB larvae at the beginning of the 5^th^ instar were counted and weighted to calculate the survival rate and the average fresh weight, respectively. For each fodder treatment, 100 larvae per group were tested. Three replicates were performed for each treatment.

### Determination of ACB biochemical responses

Nine larvae at the beginning of the 5^th^ instar stage were used for each fodder test and each test was repeated three times. In total, 27 larvae were tested for each fodder treatment. In each group, nine ACBs at the beginning of the 5^th^ instar stage were weighed and placed into a physiological saline solution at a weight ratio of 1 g tissue: 9 g physiological saline and then homogenized. The homogenates were centrifuged for 10 min at 2,500 rpm and the resulting supernatants were stored for later analysis.

The total protein contents of the ACB was determined using a total protein assay kit (with the standard BCA method; Nanjing Jiancheng, Nanjing, China). The enzyme activity of CAT was measured using a CAT assay kit (Ultraviolet) (Nanjing Jiancheng, Nanjing, China) with hydrogen peroxide (H_2_O_2_) as a substrate. The formation of H_2_O_2_–ammonium molybdate complex was monitored by the change in absorbance at 405 nm. One unit of CAT activity was defined as the amount of CAT that decomposes 1 µM H_2_O_2_ per second per mg protein. The POD and SOD activities were evaluated using guaiacol and nitroblue tetrazolium assays, respectively. POD activity was assayed by the change of absorbance at 470 nm after guaiacol oxidation in the presence of H_2_O_2_ as described previously^36^. One unit of POD activity was considered as that which oxidized 1 mg substrate per minute per mg protein. The activity of SOD was determined spectrophotometrically by measuring its ability to inhibit the reduction of nitroblue tetrazolium (NBT) according to the method of Beyer and Fridovich^37^. The amount of SOD required to inhibit 50% of the initial reduction of NBT under light was defined as one unit. GST and AChE were also measured using a Micro Glutathione S-transferase (GST) Assay Kit and a Micro Acetylcholinesterase (AchE) Assay Kit, respectively, according to the manufacturer’s instructions from the Beijing Solarbio Science & Technology (Beijing, China). GST assay used 1–chlopro–2,4–dinitrobenzene (CDNB) as a substrate, its activity was determined by monitoring the increase in the absorbance at 340 nm due to GSH–CDNB conjugate accumulation. The amount of GST that catalyses the conjugation of 1 µmol/L GSH with CDNB per minute per mg protein was regarded as one unit. The activity of AchE was determined by measuring the increase in the absorbance at 412 nm caused by the formation of 5-thio-2-nitrobenzoic acid (TNB). One unit of AchE was defined as the amount that catalyses the formation of 1 nmol TNB per minute per mg protein.

### Determination of the functional diversity of ACB’s gut microflora

The larvae at the 4^th^ instar were surface-disinfected for 3 min using 75% ethanol and then rinsed with 0.8% physiological saline solution. The intestines were removed and rinsed with saline solution. After being transferred into 1.5 mL sterilized microcentrifuge tubes, 200 µL of saline was added to each tissue sample, which was then homogenized. Next, the samples were diluted to 5 mL with saline and then transferred to a Biolog EcoPlate with 150 µL per cell. Each group of borer samples used a whole plate and each experiment was repeated three times. The plates were incubated for 168 h in a 30 °C incubator.

The metabolic capabilities and functional diversity of the gut microbial communities were assessed by Biolog assays (Biolog, Hayward, CA, USA)^38^. Each Biolog EcoPlate contains four types of carbon substrates, sugars and their derivatives, fatty acids and their derivatives, amino acids and their derivatives and secondary metabolites^39^. The absorbance of each well at 590 nm after 0-, 24-, 48-, 72-, 96-, 120-, 144- and 168 h incubations were read to calculate the average well color development (AWCD) value. AWCD = ∑(A_i_ − A_A1_)/n, where A_i_ indicates the absorbance for the i^th^ cell, A_A1_ indicates the absorbance for cell A_1_ and n indicates the number of substrates. When A_i_ − A_A1_ was negative, 0 was used in the formula.

The diversity index analysis of the gut microbial community was calculated after 96 h incubation^40^. We used three diversity indices, H, D and U, to evaluate the species richness, dominance and homogeneity, respectively, using the following three equations^41^:

$${\rm{H}}=-\sum {{\rm{P}}}_{{\rm{i}}}{\cdot \mathrm{lnP}}_{{\rm{i}}};$$

$${\rm{D}}=1-\sum {({{\rm{P}}}_{{\rm{i}}})}^{{\rm{2}}};$$

and

$${\rm{U}}={(\sum {{\rm{n}}}_{{\rm{i}}}^{2})}^{1/2},$$

where P_i_ indicates the proportion of the relative absorbance ratio of the i^th^ cell to the sum of the plate total relative absorbance; n_i_ indicates the relative absorbance of the i^th^ cell^40,42^.

### Statistical analysis

All the data obtained in this study were analysed statistically. The significances of different fodder treatments in one generation were analysed by multiple comparisons of the Duncan’s new multiple range test^43^. The significances of different fodder treatments in three generations were tested using two-way ANOVA with GraphPad Prism 5.0 software (GraphPad Software, La Jolla, CA, USA). The relationships among different samples based on discriminative carbon sources were determined using PCA analyses (Canoco, version 4.5).

## Electronic supplementary material

Supplementary Table 1

Supplementary Table 2

Supplementary Table 3

Supplementary Table 4
