# Supplementary material for: Effects of Phytase Transgenic Maize on the Physiological and Biochemical Responses and the Gut Microflora Functional Diversity of Ostrinia furnacalis
Source: Sci Rep. 2018 Mar 13;8:4413. doi: 10.1038/s41598-018-22223-x (PMC5849690; doi:10.1038/s41598-018-22223-x)
Supplement: Supplementary file 1 — Supplementary Table 1 [file 41598_2018_22223_MOESM1_ESM.pdf]

Effects of Phytase Transgenic Maize on the Physiological and Biochemical Responses and the Gut Microflora Functional Diversity of *Ostrinia furnacalis*

Xiao Hui Xu, Yinghui Guo, Hongwei Sun, Fan Li, Shuke Yang, Rui Gao and Xingbo Lu*

Supplementary Table 1 Two-way ANOVA analysis of physiological and biochemical indices of Asian corn borer larvae fed different fodders in three generations

Supplementary Table 1a Two-way ANOVA analysis of survival rates for Asian corn borer larvae fed different fodders in three generations

Effect	*F*	*P* value	Significant?
Fodder*Generation	2.268	0.1222	No
Generation	2.175	0.1563	No
Fodder	1.106	0.3901	No

Supplementary Table 1b Two-way ANOVA analysis of weights for Asian corn borer larvae fed different fodders in three generations

Effect	*F*	*P* value	Significant?
Fodder*Generation	3.424	0.0435	Yes
Generation	3.418	0.0668	No
Fodder	0.1506	0.8634	No

Supplementary Table 1c Two-way ANOVA analysis of total protein contents for Asian corn borer larvae fed different fodders in three generations

Effect	*F*	*P* value	Significant?
Fodder*Generation	0.09015	0.9838	No
Generation	0.1787	0.8385	No
Fodder	0.5578	0.5995	No

Supplementary Table 1d Two-way ANOVA analysis of catalase activities for Asian corn borer larvae fed different fodders in three generations

Effect	*F*	*P* value	Significant?
Fodder*Generation	0.6999	0.6067	No
Generation	0.2516	0.7816	No
Fodder	0.9044	0.4536	No

Supplementary Table 1e Two-way ANOVA analysis of peroxidase activities for Asian corn borer larvae fed different fodders in three generations

Effect	*F*	*P* value	Significant?
Fodder*Generation	0.6251	0.6536	No
Generation	1.982	0.1804	No
Fodder	2.079	0.2061	No

Supplementary Table 1f Two-way ANOVA analysis of superoxide dismutase activities for Asian corn borer larvae fed different fodders in three generations

Effect	*F*	*P* value	Significant?
Fodder*Generation	0.1987	0.9343	No
Generation	2.243	0.1487	No
Fodder	0.2748	0.7688	No

Supplementary Table 1g Two-way ANOVA analysis of glutathione S-transferase activities for Asian corn borer larvae fed different fodders in three generations

Effect	*F*	*P* value	Significant?
Fodder*Generation	0.2671	0.8935	No
Generation	0.6216	0.5535	No
Fodder	0.1004	0.906	No

Supplementary Table 1h Two-way ANOVA analysis of acetylcholinesterase activities for Asian corn borer larvae fed different fodders in three generations

Effect	*F*	*P* value	Significant?
Fodder*Generation	1.588	0.2405	No
Generation	23.07	< 0.0001	Yes
Fodder	0.8829	0.4612	No
