# Supplementary material for: Effects of Phytase Transgenic Maize on the Physiological and Biochemical Responses and the Gut Microflora Functional Diversity of Ostrinia furnacalis
Source: Sci Rep. 2018 Mar 13;8:4413. doi: 10.1038/s41598-018-22223-x (PMC5849690; doi:10.1038/s41598-018-22223-x)
Supplement: Supplementary file 2 — Supplementary Table 2 [file 41598_2018_22223_MOESM2_ESM.pdf]

Effects of Phytase Transgenic Maize on the Physiological and Biochemical Responses and the Gut Microflora Functional Diversity of *Ostrinia furnacalis*

Xiao Hui Xu, Yinghui Guo, Hongwei Sun, Fan Li, Shuke Yang, Rui Gao and Xingbo Lu*

Supplementary Table 2 Two-way ANOVA analysis of AWCD changes of Asian corn borer larvae gut microflora in three generations.

Supplementary Table 2a AWCD at 0 h

Effect	*F*	*P* value	Significant?
Fodder*Generation	0.2938	0.8764	No
Generation	0.09734	0.908	No
Fodder	2.925	0.1298	No

Supplementary Table 2b AWCD at 24 h

Effect	*F*	*P* value	Significant?
Fodder*Generation	0.3432	0.8437	No
Generation	11.23	0.0018	Yes
Fodder	0.1733	0.8449	No

Supplementary Table 2c AWCD at 48 h

Effect	*F*	*P* value	Significant?
Fodder*Generation	0.1441	0.9622	No
Generation	63.17	< 0.0001	Yes
Fodder	0.01883	0.9814	No

Supplementary Table 2d AWCD at 72 h

Effect	*F*	*P* value	Significant?
Fodder*Generation	1.523	0.2571	No
Generation	41.2	< 0.0001	Yes
Fodder	0.8306	0.4803	No

Supplementary Table 2e AWCD at 96 h

Effect	*F*	*P* value	Significant?
Fodder*Generation	0.5037	0.7339	No
Generation	66.87	< 0.0001	Yes
Fodder	0.2532	0.7842	No

Supplementary Table 2f AWCD at 120 h

Effect	*F*	*P* value	Significant?
Fodder*Generation	0.5267	0.7184	No
Generation	47.97	< 0.0001	Yes
Fodder	0.5435	0.6068	No

Supplementary Table 2g AWCD at 144 h

Effect	*F*	*P* value	Significant?
Fodder*Generation	1.119	0.3925	No
Generation	64.19	< 0.0001	Yes
Fodder	1.016	0.4169	No

Supplementary Table 2h AWCD at 168 h

Effect	*F*	*P* value	Significant?
Fodder*Generation	1.15	0.3798	Yes
Generation	35.23	< 0.0001	Yes
Fodder	0.416	0.6773	No
