# Supplementary material for: Effects of Phytase Transgenic Maize on the Physiological and Biochemical Responses and the Gut Microflora Functional Diversity of Ostrinia furnacalis
Source: Sci Rep. 2018 Mar 13;8:4413. doi: 10.1038/s41598-018-22223-x (PMC5849690; doi:10.1038/s41598-018-22223-x)
Supplement: Supplementary file 3 — Supplementary Table 3 [file 41598_2018_22223_MOESM3_ESM.pdf]

Effects of Phytase Transgenic Maize on the Physiological and Biochemical Responses and the Gut Microflora Functional Diversity of *Ostrinia furnacalis*

Xiao Hui Xu, Yinghui Guo, Hongwei Sun, Fan Li, Shuke Yang, Rui Gao and Xingbo Lu*

Supplementary Table 3 Two-way ANOVA analysis of carbon sources utilization by Asian corn borer larvae gut microflora in three generations.

Supplementary Table 3a Sugars and their derivatives

Effect	*F*	*P* value	Significant?
Fodder*Generation	8.028	0.0022	Yes
Generation	442.5	< 0.0001	Yes
Fodder	0.01555	0.9846	No

Supplementary Table 3b Fatty acids and their derivatives

Effect	*F*	*P* value	Significant?
Fodder*Generation	7.904	0.0023	Yes
Generation	477.3	< 0.0001	Yes
Fodder	0.9091	0.452	No

Supplementary Table 3c Amino acids and their derivatives

Effect	*F*	*P* value	Significant?
Fodder*Generation	1.593	0.2393	No
Generation	258.6	< 0.0001	Yes
Fodder	1.756	0.251	No

Supplementary Table 3d Intermediate and secondary metabolites

Effect	*F*	*P* value	Significant?
Fodder*Generation	36.2	< 0.0001	Yes
Generation	2646	< 0.0001	Yes
Fodder	0.2746	0.769	No
