# Supplementary material for: Effects of Phytase Transgenic Maize on the Physiological and Biochemical Responses and the Gut Microflora Functional Diversity of Ostrinia furnacalis
Source: Sci Rep. 2018 Mar 13;8:4413. doi: 10.1038/s41598-018-22223-x (PMC5849690; doi:10.1038/s41598-018-22223-x)
Supplement: Supplementary file 4 — Supplementary Table 4 [file 41598_2018_22223_MOESM4_ESM.pdf]

Effects of Phytase Transgenic Maize on the Physiological and Biochemical Responses and the Gut Microflora Functional Diversity of *Ostrinia furnacalis*

Xiao Hui Xu, Yinghui Guo, Hongwei Sun, Fan Li, Shuke Yang, Rui Gao and Xingbo Lu*

Supplementary Table 4 Two-way ANOVA analysis of Shannon's, Simpson and McIntosh indices for Asian corn borer larvae fed different fodders in three generations

Supplementary Table 4a Two-way ANOVA analysis of Shannon's diversity indices for Asian corn borer larvae fed different fodders in three generations

Effect	*F*	*P* value	Significant?
Fodder*Generation	0.8498	0.5205	No
Generation	36.63	< 0.0001	Yes
Fodder	1.291	0.3416	No

Supplementary Table 4b Two-way ANOVA analysis of Simpson indices for Asian corn borer larvae fed different fodders in three generations

Effect	*F*	*P* value	Significant?
Fodder*Generation	0.2934	0.8767	No
Generation	55.95	< 0.0001	Yes
Fodder	2.492	0.163	No

Supplementary Table 4c Two-way ANOVA analysis of McIntosh indices for Asian corn borer larvae fed different fodders in three generations

Effect	*F*	*P* value	Significant?
Fodder*Generation	3.331	0.0471	Yes
Generation	499.1	< 0.0001	Yes
Fodder	0.1266	0.8834	No
